# Polymorphisms of *GSTP1*, *ERCC2* and *TS*-3′UTR are associated with the clinical outcome of mFOLFOX6 in colorectal cancer patients

**DOI:** 10.3892/ol.2013.1467

**Published:** 2013-07-15

**Authors:** KENSUKE KUMAMOTO, KEIICHIRO ISHIBASHI, NORIMICHI OKADA, YUSUKE TAJIMA, KOUKI KUWABARA, YOICHI KUMAGAI, HIROYUKI BABA, NORIHIRO HAGA, HIDEYUKI ISHIDA

**Affiliations:** Department of Digestive Tract and General Surgery, Saitama Medical Center, Saitama Medical University, Kawagoe, Saitama 350-8550, Japan

**Keywords:** FOLFOX, colorectal cancer, polymorphism

## Abstract

The aim of the current study was to examine whether polymorphisms in drug metabolism genes have any clinical impact on patients treated with 5-fluorouracil (FU)/oxaliplatin for metastatic colorectal cancer (MCRC). In total, 63 patients with MCRC were recruited and treated with a modified FOLFOX6 (mFOLFOX6) treatment as a first-line chemotherapy. Polymorphisms in five drug metabolism genes and two DNA-repair genes were assessed in these patients using polymerase chain reaction (PCR), a PCR restriction fragment length polymorphism (PCR-RFLP) technique or invader techniques. These included a 28-bp tandem repeat in the 5′-untranslated region (UTR) and 6-bp deletions in the 3′-UTR of thymidylate synthase (*TS*), methylenetetrahydrofolate reductase (*MTHFR*; Ala677Val), glutathione S-transferase π (*GSTP1*; IIe105Val), GST θ1 (*GSTT1*; deletion) and GST μ1 (*GSTM1*; deletion) and the two DNA-repair genes, excision repair cross-complementing-1 (*ERCC1*; Asp118Asn) and *ERCC2* (Lys751Gln). The correlation between these polymorphisms and the clinical outcome, including drug response, progression-free survival (PFS), overall survival (OS) and the incidence of peripheral neuropathy, were evaluated. Patients with the *GSTP1*-105 A/A genotype had poor responses to mFOLFOX6 treatment compared with those with the *GSTP1*-105 A/G and G/G genotypes (P=0.01). The median PFS of patients with the *ERCC2-*751 A/A genotype tended to be longer than that of patients with the *ERCC2*-751 A/C genotype (P=0.05). Patients with the *TS*-3′-UTR −6/−6 genotype had a significantly longer OS compared with patients with other genotypes (P=0.003). A statistically significant association between the incidence of peripheral neuropathy higher than grade 2 and the *GSTP1*-105 (P=0.03) and *GSTM1* genotypes (P=0.02) was identified by multivariate logistic regression analyses. Results demonstrated that polymorphisms in *GSTP1*-105, *ERCC2*-751 and the 3′-UTR of *TS* may be a statistically significant predictors of clinical outcome. *GSTP1*-105 and *GSTM1* genotypes may be useful markers of severe peripheral neuropathy in MCRC patients treated with 5-FU/oxaliplatin as first-line chemotherapy.

## Introduction

Advances in chemotherapeutic regimens for metastatic colorectal cancer (MCRC) patients, including FOLFOX treatment comprising of a combination of 5-fluorouracil (FU)/leucovorin (LV) and oxaliplatin, have improved overall survival (OS) (1,2). It has been reported that the efficacy rate of FOLFOX treatment varies between 20 and 50% in MCRC patients (1,3–5). Individuals who receive chemotherapy commonly suffer from side effects, including myelosuppression, nausea, diarrhea and peripheral neuropathy (1–3). Numerous patients undergoing FOLFOX treatment have complained of oxaliplatin-induced peripheral neuropathy. Therefore, several markers for predicting the efficacy of FOLFOX treatment have been investigated to identify patients with favorable treatment prognoses. Gene expression analysis, associated with the metabolism of 5-FU and oxaliplatin, has been intensively studied (6–9). It has been reported that thymidylate synthase (*TS)* and thymidine phosphorylase (*TP*) mRNA expression levels are useful markers for predicting the efficacy of FOLFOX treatment in CRC patients with liver metastasis (10). In addition, advances in molecular biology indicate that a number of drug metabolism genes have polymorphisms that alter levels of expression. Among these, polymorphisms have been identified in *TS*, excision repair cross-complementing-1 (*ERCC1)* and *ERCC2*, glutathione S-transferase π (*GSTP1)*, GST θ1 (*GSTT1)*, GST μ1 (*GSTM1)* and methylenetetrahydrofolate reductase (*MTHFR)*, which exert functions in drug metabolism and antidotal effects on the 5-FU and oxaliplatin pathways. Studies have demonstrated that specific polymorphisms of these genes are associated with the efficacy of FOLFOX treatment in MCRC patients (11–14).

*TS* and *MTHFR* are associated with the metabolism of 5-FU, indicating that their altered expression affects the response to 5-FU-based chemotherapy. The enzyme product of *TS* is critical for catalyzing the methylation of deoxyuridine-5′-monophosphate to deoxythymidine-5′-monophosphate in *de novo* DNA synthesis. Fluorodeoxyuridine monophosphate (FdUMP), the metabolic product of 5-FU, forms complexes with TS and 5,10-methylenetetrahydrofolate, resulting in the inhibition of DNA synthesis. Two polymorphisms have been identified in *TS,* a variable length tandem repeat polymorphism in the 5′-untranslated region (UTR) that consists of two or three 28-bp repeated sequences and a 6-bp insertion/deletion (6+/6−) in the 3′-UTR. A number of studies have described correlations between genotype patterns of polymorphisms in *TS* and the efficacy of FOLFOX treatment for MCRC patients (11–14). However, current evidence is insufficient to confirm a statistically significant correlation. MTHFR is important for folate metabolism and catalyzing the conversion of 5,10-methylenetetrahydrofolate to 5-methyltetrahydrofolate. Two important polymorphisms in *MTHFR*, C677T and A1298C, have been studied (12–14) and have been identified to affect the enzyme activity of MTHFR (15,16), leading to the accumulation of 5,10-methylenetetrahydrofolate and the enhanced sensitivity of 5-FU by forming complexes with TS and FdUMP. Studies have described the correlation among these polymorphisms and the efficacy of FOLFOX treatment for MCRC patients (12,14).

The expression levels of *ERCC1*, *ERCC2*, *GSTP1*, *GSTT1* and *GSTM1* have been hypothesized to be associated with the efficacy of platinum compounds, including cisplatin and oxaliplatin. *ERCC-1* and *-2* are involved in DNA repair and tolerance of DNA damage through the nucleotide excision repair pathway. The enhanced expression of these proteins may lead to the resistance to platinum drugs. A common C to T transition at codon 118 of *ERCC1* has been shown to increase expression in patients with the T/T genotype compared with patients with the C/T or C/C genotypes (17), despite the T/T polymorphism producing the identical amino acid, asparagine. Studies have revealed that patients with the T/T genotype have a poor outcome compared with patients with the C/T or C/C genotypes (13). During oxaliplatin-based chemotherapy, the prognosis for MCRC patients with the C/C genotype is more encouraging than that of patients with other genotypes (11,18,19). The xeroderma pigmentosum group D (XPD) gene, *ERCC2*, has three common polymorphisms in codons 156, 312 and 751. The polymorphism at codon 751 (A>C: Lys>Gln) is associated with the clinical outcome of MCRC patients receiving FOLFOX treatment (13,20).

The GST family includes at least five subclasses with major biological roles in the detoxification of genotoxic compounds. *GSTP1*, *GSTT1* and *GSTM1* genotypes have been extensively studied for drug response, including oxaliplatin-based treatment (21–23). A single nucleotide polymorphism at codon 105 (A>G: Ile>Val) of *GSTP1* affects enzyme activity (24). Several studies have demonstrated that among MCRC patients receiving oxaliplatin-based treatment, patients with *GSTP1*-105 A/G and G/G genotypes have a more favorable outcome compared with patients with the *GSTP1*-105 A/A genotype (21–23).

In the present study, correlations were identified between the polymorphism patterns of *TS*, *MTHFR*, *ERCC1*, *ERCC2*, *GSTP1, GSTT1* and *GSTM1* and the clinical outcome, including the incidence of peripheral neuropathy, in Japanese MCRC patients who were treated with modified FOLFOX6 (mFOLFOX6).

## Materials and methods

### Patients and clinical procedures

The current study was performed in accordance with the ethical guidelines for clinical studies with approval from the institutional ethics committee. Informed consent was obtained from all individuals.

The subjects included 63 CRC patients (22 females and 41 males) who received mFOLFOX6 treatment as first-line chemotherapy between 2005 and 2009. The mFOLFOX6 regimen was comprised of intravenous infusions of oxaliplatin (85 mg/m^2^) and LV (200 mg/m^2^) for 2 h, followed by a rapid intravenous bolus infusion of 5-FU (400 mg/m^2^) for 5 min and a continuous intravenous infusion of 5-FU (2,400 mg/m^2^) for 46 h. This regimen was repeated every 2 weeks. Table I presents the patient characteristics. The median age of the patients was 65 years old (range, 32–84 years old). The primary site was the colon/rectosigmoid in 43 patients and the rectum in 20 patients. Performance status (PS), determined according to the method of the Eastern Cooperative Oncology Group was 0 in 39 patients, 1 in 21 patients and 2 in 3 patients. The target lesions were located in the liver of 43 patients, the lungs of 18 patients, the peritoneum of 13 patients and the lymph nodes of 16 patients, while in 7 patients the target lesions were detected in other locations. The median number of oxaliplatin doses was 10 (range, 4–39) and the median relative dose intensity of oxaliplatin was 75% (range, 28.1–100%). The response to mFOLFOX6 treatment was evaluated during 4–6 courses of treatment according to the Response Evaluation Criteria in Solid Tumors (version 1.1) (25). Complete response was observed in 3 patients, partial response in 23 patients, stable disease in 24 patients and progressive disease in 13 patients. Adverse events were graded according to the Common Terminology Criteria for Adverse Events (version 3.0). When an adverse event of >grade 3 severity occurred, mFOLFOX6 therapy was suspended until the severity of the reaction improved to <grade 2. When mFOLFOX6 therapy was resumed, doses of oxaliplatin were reduced to 70–80% of the previous dose.

Of the 63 patients with CRC, 44 received folinic acid/5-FU/irinotecan (FOLFIRI) either with (n=15) or without bevacizumab (n=29) as a second-line chemotherapy treatment. The FOLFIRI regimen comprised of intravenous infusions of irinotecan (150 mg/m^2^) and LV (200 mg/m^2^) for 2 h, followed by a rapid intravenous bolus infusion of 5-FU (400 mg/m^2^) for 5 min and a continuous intravenous infusion of 5-FU (2,400 mg/m^2^) for 46 h, administered every 2 weeks. A total of 16 patients were observed without administration of additional treatment.

### DNA extraction and analysis of polymorphisms

Genomic DNA was extracted from 23 blood samples and 40 normal colonic mucosae from each enrolled patient using the QIAamp DNA Blood and QIAamp DNA Mini kits (Qiagen, Tokyo, Japan). Polymorphisms were analyzed by polymerase chain reaction (PCR), a PCR restriction fragment length polymorphism (PCR-RFLP) technique and a PCR-invader method. Primer sequences and restriction enzymes of all genes examined are presented in Table II.

### Statistical analysis

Continuous data are presented as the median and range. Mann-Whitney U, Fisher’s exact probability and χ^2^ tests were used where applicable. A survival analysis was conducted using the Kaplan-Meier method. The log-rank test was used to determine the significance of the survival curves. The OS period was calculated between the time of surgery and the date of mortality of any cause. OS was censored from the time of the individuals last visit to the hospital or December 2010, depending on which was the first event. Logistic regression was used to determine independent predictors of adverse events. P<0.05 was considered to indicate a statistically significant difference. All statistical analyses were performed using a statistical software package (StatFlex ver.6.0; Artech, Osaka, Japan).

## Results

### Correlation between polymorphisms in TS, MTHFR, ERCC1, ERCC2, GSTP1, GSTT1 and GSTM1 and the response rate to mFOLFOX6 treatment

Polymorphisms of *GSTP1*-105 were shown to significantly correlate with the efficacy of mFOLFOX6 treatment (Table III). The frequencies of *GSTP1*-105 A/A, A/G and G/G genotypes were 70, 25 and 3%, respectively. In the responder group, fewer patients expressed the *GSTP1*-105 A/A genotype than the *GSTP1*-105 A/G or G/G genotypes (P=0.01). No significant differences were identified between the polymorphisms of other genes and the efficacy of mFOLFOX6 treatment.

### Correlation between polymorphisms in TS, MTHFR, ERCC1, ERCC2, GSTP1, GSTT1 and GSTM1 and PFS and OS in MCRC patients treated with mFOLFOX6

**Figure 1:**
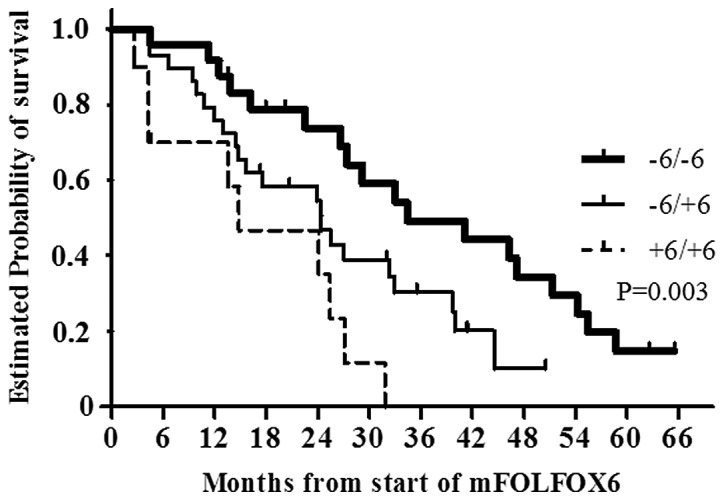
Overall survival (OS) in metastatic colorectal cancer patients with *TS*-3′-UTR −6/−6 (n=24), −6/+6 (n=29) and +6/+6 (n=10) genotypes. The median OS of patients with the *TS*-3′-UTR −6/−6, −6/+6 and +6/+6 genotypes was 34.4, 24.4 and 14.8 months, respectively. TS, thymidylate synthase; UTR, untranslated region.

Frequencies of the *ERCC2*-751 A/A, A/C and C/C genotypes were 92, 8 and 0%, respectively (Table III). The median PFS of patients with the *ERCC2*-751 A/A genotype was longer than that of patients with the *ERCC2*-751 A/C genotype (10.3 and 6.1 months, respectively; P=0.05). There was no correlation between polymorphisms of other genes and PFS. The median OS of the patients with the *TS*-3′-UTR −6/−6 (n=24), −6/+6 (n=29) and +6/+6 (n=10) genotypes was 34.4, 24.4 and 14.8 months, respectively. The OS of the patients with the *TS*-3′-UTR −6/−6 genotype was significantly longer compared with that of the patients with other genotypes (P=0.003; Fig. 1).

### Correlation between polymorphisms in TS, MTHFR, ERCC1, ERCC2, GSTP1, GSTT1 and GSTM1 and incidence of peripheral neuropathy in patients treated with mFOLFOX6

The incidence of peripheral neuropathy of grades 2 (n=42) and 3 (n=2) was found to significantly correlate with the *GSTP1*-105 (P=0.05) and *GSTM1* (P=0.03) genotypes, as identified by univariate regression analyses (Table IV). Peripheral neuropathy occurred in the majority of patients with the *GSTP1*-105 A/G and G/G genotypes compared with patients with the *GSTP1*-105 A/A genotype. Individuals who were *GSTM1*-negative also had peripheral neuropathy, whereas individuals who were *GSTM1*-positive did not. A statistically significant correlation between the incidence of peripheral neuropathy higher than grade 2 and the *GSTP1*-105 (P=0.03) and *GSTM1* (P=0.02) genotypes was determined using multivariate regression analysis.

## Discussion

In the present study, specific polymorphisms of genes involved in 5-FU/oxaliplatin metabolism were demonstrated to be significantly associated with the clinical outcome of Japanese MCRC patients who received first-line chemotherapy with mFOLFOX6. The response to mFOLFOX6 treatment in patients with the *GSTP1*-105 A/G and G/G genotypes was significantly improved compared with that of patients with the *GSTP1*-105 A/A genotype. In addition, the *ERCC2*-751 and *TS*-3′-UTR genotypes were shown to significantly correlate with PFS and OS, respectively. The results indicated that polymorphisms in the oxaliplatin-associated genes, *GSTP1*-105 and *ERCC2*-751, were hypothesized to be important for the prediction of primary clinical outcome, including drug responses and PFS, for MCRC patients treated with mFOLFOX6. Second- and third-line chemotherapy regimens also affected OS. Of the 63 patients, 44 were treated with FOLFIRI following the FOLFOX regimen and 5-FU treatment continued throughout. Therefore, it is possible that polymorphisms in the genes involved in 5-FU metabolism contribute to OS in long-term observations.

Previous studies have revealed that *GSTP1*-105 genotypes are associated with the clinical outcome of MCRC patients who receive 5-FU/oxaliplatin as first-line chemotherapy (11–13,21–23). As *GSTP1* expression is enhanced in CRC (26) it has been hypothesized to be involved in the resistance to platinum compounds (27). The enzyme activity of the *GSTP1*-105 A/G and G/G genotypes is lower than that of the *GSTP1*-105 A/A genotype (24). In addition, clinical assessments of the correlation between the *GSTP1* genotype and the clinical outcome in MCRC patients treated with 5-FU/oxaliplatin appears to be consistent with basic studies. In the present study, patients with the *GSTP1*-105 A/G and G/G genotypes were revealed to have a significantly improved response to mFOLFOX6 treatment when compared with patients with the *GSTP1*-105 A/A genotype. Previous studies have indicated that the *GSTP1*-105 A/G and G/G genotypes are significantly more common than the *GSTP1*-105 A/A genotype among patients who respond to 5-FU/oxaliplatin treatment (22,23). In addition to drug response, several studies have demonstrated that MCRC patients with the *GSTP1*-105 A/G and G/G genotypes have favorable outcomes following oxaliplatin-based treatment compared with patients with the *GSTP1*-105 A/A genotype (11,21). This tendency was also recognized in the results of the current study. The frequencies of the *GSTP1*-105 A/A, A/G and G/G polymorphisms were 70, 25 and 3%, respectively, in the Japanese population, which is similar to frequencies reported in other Asian populations, including Chinese and Taiwanese (11,22,24). In American and European populations, there is an almost equal frequency of *GSTP1*-105 A/A and A/G carriers, which combine to make a total of ~90% of all patients. By contrast, the frequency of the *GSTP1*-105 G/G genotype is ~10% in these populations (11–13,21,24). Regardless of ethnic differences, the association of the *GSTP1*-105 genotype with the clinical outcome is consistent among all MCRC patients who receive 5-FU/oxaliplatin as first-line chemotherapy.

In addition to *GSTP1* and *ERCC-1* and *-2*, members of the nucleotide excision repair pathway are involved in repair and tolerance of DNA damage and also encode key enzymes for oxaliplatin metabolism. Several studies have demonstrated that the *ERCC1*-118 and *ERCC2*-751 genotypes are associated with the clinical outcome of MCRC patients receiving oxaliplatin-based treatment (11,13,18–20). In the present study, the *ERCC2*-751 genotypes were significantly associated with PFS, whereas no significant difference was identified between the ERCC1-118 genotype and the clinical outcome. The PFS of the patients with the *ERCC2*-751 A/A genotype was longer than that of patients with the *ERCC2*-751 A/C genotype, and this was consistent with previous studies (11,13,20). The distribution of *ERCC2*-751 polymorphisms clearly differs between Asian and Western individuals. Among Asians, the frequencies of the *ERCC2*-751 A/A, A/C and C/C genotypes are 84–92, 8–16 and 0%, respectively (11,22), whereas among Americans and Europeans the frequencies are 25–38, 50–61 and 11–15%, respectively (11–13,20,22,23). There is a high possibility that the majority of Asians carry the *ERCC2*-751 A/A genotype, leading to promising outcomes of oxaliplatin-based chemotherapy.

A statistically significant association between *TS*-3′-UTR genotypes and OS was identified in the current study. Treatment with FOLFIRI was also administered as second-line chemotherapy to ~70% of patients receiving mFOLFOX6 treatment, and hence, the patients with increased survival rates were exposed to 5-FU for a long time. Numerous studies have indicated that MCRC patients with lower *TS* expression have a favorable outcome following 5-FU-based chemotherapy compared with patients with high *TS* expression (10,28). A previous study revealed that *TS* mRNA expression in rectal cancer patients with the *TS*-3′-UTR −6/−6 and −6/+6 genotypes was significantly lower compared with patients with the *TS*-3′-UTR +6/+6 genotype, resulting in a favorable outcome following neoadjuvant 5-FU-based chemoradiation (29). Among CRC patients receiving 5-FU-based adjuvant treatment, the OS of patients with the *TS*-3′-UTR −6/−6 genotype was significantly longer compared with that of patients with other genotypes (30). Although there are various types of cancer, an encouraging association between clinical outcome and the *TS*-3′-UTR −6/−6 genotype has been identified in Asian gastric cancer patients receiving mFOLFOX6 treatment (31). Several studies have reported that there is no correlation between the *TS*-3′-UTR genotype and the clinical outcome of MCRC patients receiving 5-FU/oxaliplatin treatment (12–14,23). The frequency distribution of the *TS*-3′-UTR genotype may lead to discrepancies in the clinical outcome. In the current study, the frequencies of the *TS*-3′-UTR −6/−6, −6/+6 and +6/+6 genotypes were 38, 46 and 16%, respectively, while in the USA and Europe these genotypes are 10–16, 37-5 and 33–53%, respectively (11–13,23). Further studies may be required to clarify the association between these differences in ethnicity and the efficacy of anti-cancer drugs.

While mFOLFOX6 treatment improves the survival rate of MCRC patients, adverse events, including myelosuppression, nausea, diarrhea and peripheral neuropathy, are common. In particular, peripheral neuropathy, caused by cumulative administration of oxaliplatin, directly affects the quality of life and is a major reason for the discontinuation of oxaliplatin chemotherapy. Thus, predictive markers of peripheral neuropathy are required for prospective evaluations. In agreement with previous studies, the incidence of peripheral neuropathy higher than grade 2 was identified to significantly correlate with the *GSTP1*-105 and *GSTM1* genotypes (13,22). Notably, peripheral neuropathy in patients with the *GSTP1*-105 A/G and G/G genotypes was of greater intensity compared with that of patients with the *GSTP1*-105 A/A genotype. A statistically significant correlation was identified between the *GSTP1*-105 genotype and the clinical outcome. Therefore, the *GSTP1*-105 polymorphism may serve as a double-edged marker for predicting response to 5-FU/oxaliplatin treatment and the intensity of oxaliplatin-associated peripheral neuropathy.

In the present study, the association among gene polymorphisms that affect the metabolism of 5-FU oxaliplatin and the clinical outcome in Japanese patients with MCRC was identified. Ethnic differences in the frequency distribution of polymorphisms, which preclude the extrapolation of clinical studies between Western and Asian populations, were also identified. Therefore, the present study is likely to improve chemotherapy for individuals of Asian descent. Consistent with studies in Western patients, the polymorphisms of *GSTP1*-105, *ERCC2*-751 and the 3′-UTR of *TS* were associated with the clinical outcome of FOLFOX treatment in Japanese MCRC patients. Therefore, these polymorphisms may be significant predictors of clinical outcome globally. However, *GSTP1*-105 and *GSTM1* genotypes may be more useful as markers for severe oxaliplatin-induced peripheral neuropathy in Japanese patients compared with Western patients.

## Figures and Tables

**Table I:** Patient characteristics.

Parameter	Value
Total patients, n	63
Gender, males:females	41:22
Age, years^a^	65 (32–84)
Location, n	
Colon/rectosigmoid	43
Rectum	20
Performance status, n	
0	39
1	21
2	3
Number of target organ(s)	
1	26
>2	37
Target organ, n	
Liver	43
Lung	18
Lymph node	16
Peritoneum	13
Others	7
Cycles of mFOLFOX6 therapy, n^a^	10.0 (4–39)
Relative dose intensity, %^a^	75.0 (28.1–100)
Response, n	
CR	3
PR	23
SD	24
PD	13
Second line chemotherapy, n	
FOLFIRI	29
FOLFIRI + bevacizumab	15
Other	3
Best supportive care	16

a Median (range).

CR, complete response; PR, partial response; SD, stable disease; PD, progressive disease; FOLFIRI, folinic acid/5-FU/irinotecan.

**Table II:** Characteristics of polymorphisms with primer sequences and restriction enzymes.

Site	Polymorphism	Genotype	Restriction enzymes	Primers	Detection method
*TS* 5′-UTR	VNTR	2R or 3R alleles		5′-AGGCGCGCGGAAGGGGTCCT-3′ 5′-TCCGAGCCGGCCACAGGCAT-3′	PCR
*TS* 3′-UTR	6 bp insertion/deletion	6+/6−	*Dra*l	5′-CAAATCTGAGGGAGCTGAGT-3′ 5′-CAGATAAGTGGCAGTACAGA-3′	PCR-RFLP
*MTHFR* (exon 4)	SNP	C/T, Ala677Val	*Hin*fi	5′-TGAAGGAGATGTCTGCGGGA-3′ 5′-AGGACGGTGCGGTGAGAGTG-3′	Invader method
*ERCC1* (exon 4)	SNP	C/T, Asn118Asn	*Mae*ll	5′-GAGAGGGCTGAGCTGGAGACAG-3′ 5′-CCAGCACATAGTCGGGAATTACGTC-3′	PCR-RFLP
*ERCC2* (exon 23)	SNP	A/C, Lys751Gln	*Mbo*ll	5′-CAGGTGAGGGGGACATCTG-3′ 5′-CTCTCCCTTTCCTCTGTTC-3′	PCR-RFLP
*GSTP1* (exon 5)	SNP	A/G, IIe105Val	*Msp*l	5′-ACCCCAGGGCTCTATGGGAA-3′ 5′-TGAGGGCACAAGCCCCT-3′	PCR-RFLP
*GSTT1*	Deletion	±^a^		5′-TTCCTTACTGGTCCTCCTCACATCTC-3′ 5′-TCACCGGATCATGGCCAGCA-3′	PCR
*GSTM1*	Deletion	±^a^		5′-GAACTCCCTGAAAAGCTAAAGC-3′ 5′-GTTGGGCTCAAATATACGGTGG-3′	PCR

a Genotype was defined as positive if at least one copy of the gene was present.

TS, thymidylate synthase; VNTR, variable number tandem repeat; 2R/3R, two/three 28-bp repeated sequences; UTR, untranslated region; MTHFR, methylenetetrahydrofolate reductase; ERCC, excision repair cross-complementing; SNP, single uncleotide polymorphism; GSTP1, glutathione S-transferase π; GSTT1, glutathione S-transferase θ1; GSTM1, glutathione S-transferase μ1; PCR, polymerase chain reaction; RFLP, restriction fragment length polymorphism.

**Table III:** Frequency of polymorphisms, response rate and median PFS and OS.

Gene	Patients n=63, n (%)	Responder, n (%)	P-value	Median PFS^b^, months	P-value	Median OS^b^, months	P-value
*TS*-5′UTR			0.11		0.56		0.650
3R/3R	46 (73)	18 (39)		8.6		27.0	
2R/3R	13 (21)	8 (62)		9.9		25.4	
2R/2R	3 (5)	0 (0)		11.1		31.8	
Unknown	1 (2)						
*TS*-3′UTR			0.93		0.48		0.003
−6/−6	24 (38)	10 (42)		11.6		34.4	
−6/+6	29 (46)	13 (45)		8.3		24.4	
+6/+6	10 (16)	3 (30)		10.7		14.8	
*MTHFR*-677			0.70		0.80		0.860
C/C	26 (41)	12 (46)		9.9		27.4	
C/T	30 (48)	11 (37)		8.1		27.0	
T/T	6 (10)	3 (50)		8.3		24.4	
Unknown	1 (2)						
*ERCC1*-118			0.71		0.63		0.380
C/C	30 (48)	11 (37)		9.9		27.4	
C/T	23 (37)	11 (48)		8.1		22.5	
T/T	10 (16)	4 (40)		8.3		32.9	
*ERCC2*-751			0.95		0.05		0.690
A/A	58 (92)	24 (41)		10.3		25.5	
A/C	5 (8)	2 (40)		6.1		29.2	
C/C	0 (0)						
*GSTP1*-105			0.05		0.41		0.260
A/A	44 (70)	14 (32)	0.01^a^	8.6		24.4	
A/G	16 (25)	11 (69)		7.8		31.1	
G/G	2 (3)	1 (50)		11.8		46.3	
Unknown	1 (2)						
*GSTT1*			0.83		0.47		0.840
Positive	30 (48)	13 (43)		8.1		25.5	
Negative	32 (51)	13 (41)		10.3		27.1	
Unknown	1 (2)						
*GSTM1*			0.73		0.89		0.480
Positive	23 (37)	9 (39)		7.4		22.5	
Negative	39 (62)	17 (44)		10.7		27.4	
Unknown	1 (2)						

a Comparison between A/A and A/G + G/G,

b Kaplan-meier methods, logrank test.

PFS, progression-free survival; OS, overall survival; TS, thymidylate synthase; VNTR, variable number tandem repeat; UTR, untranslated region; MTHFR, methylenetetrahydrofolate reductase; ERCC, excision repair cross-complementing; SNP, single uncleotide polymorphism; GSTP1, glutathione S-transferase π; GSTT1, glutathione S-transferase θ1; GSTM1, glutathione S-transferase μ1.

**Table IV:** Correlation between peripheral neuropathy and polymorphisms.

Gene	Patients, n	Patients with >Grade 2, n (%)	Univariate regression analysis			Multivariate regression analysis		
	
			OR	95% CI	P-value	OR	95% CI	P-value
*TS*-5′UTR								
3R/3R	46	32 (70)	1					
2R/2R, 2R/3R	16	11 (69)	0.962	0.281–3.289	0.95			
*TS*-3′UTR								
−6/−6	24	19 (79)	1					
−6/+6, +6/+6	39	25 (64)	0.470	0.144–1.533	0.21			
*MTHFR*-677								
C/C	26	19 (73)	1					
C/T, T/T	36	24 (67)	0.737	0.243–2.235	0.59			
*ERCC1*-118								
C/C	30	20 (67)	1					
C/T, T/T	33	24 (73)	1.333	0.453–3.921	0.60			
*ERCC2*-751								
A/A	58	41 (71)	1					
A/C, C/C	5	3 (60)	0.622	0.095–4.062	0.62			
*GSTP1*-105								
A/A	44	27 (61)	1			1		
A/G, G/G	18	16 (89)	5.037	1.027–24.712	0.05	6.084	1.150–32.175	0.03
*GSTT1*								
Positive	30	21 (70)	1					
Negative	32	22 (69)	0.943	0.320–2.778	0.92			
*GSTM1*								
Positive	23	12 (52)	1			1		
Negative	39	31 (79)	3.546	1.149–10.989	0.03	4.202	1.253–14.085	0.02

TS, thymidylate synthase; UTR, untranslated region; MTHFR, methylenetetrahydrofolate reductase; ERCC, excision repair cross-complementing; GSTP1, glutathione S-transferase π; GSTT1, glutathione S-transferase θ1; GSTM1, glutathione S-transferase μ1; OR, odds ratio; CL, confidence interval.
